# Significance of serum and pathological biomarkers in fertility-sparing treatment for endometrial cancer or atypical hyperplasia: a retrospective cohort study

**DOI:** 10.1186/s12905-021-01383-5

**Published:** 2021-06-23

**Authors:** Yiqin Wang, Rong Zhou, Xiaobo Zhang, Huixin Liu, Danhua Shen, Jianliu Wang

**Affiliations:** 1 Department of Obstetrics and Gynecology, Peking University People’s Hospital, 11th Xizhimen South Street, Xicheng District, Beijing, 100044 China; 2 Department of Pathology, Peking University People’s Hospital, Beijing, China; 3 Department of Clinical Epidemiology, Peking University People’s Hospital, Beijing, China

**Keywords:** Endometrial cancer, Endometrial atypical hyperplasia, Fertility-sparing, Insulin resistance, Progesterone receptor, Treatment efficacy

## Abstract

**Background:**

This study analyzed the changes of serum and pathological biomarkers during fertility-sparing therapy of endometrial cancer (EC) or endometrial atypical hyperplasia (EAH), to investigate their implications for early prediction of treatment efficacy.

**Methods:**

A retrospective analysis of EC or EAH patients who received fertility-sparing therapy between 2012 and 2016 was performed. Serum and endometrium sampling were obtained for each patient at three time points: at baseline, at 3–6 months' treatment and at the end of conservative treatment. Serum biomarkers including insulin resistance (HbA1c, HOMA-IR), sex hormones and thyroid hormones were measured. Meanwhile expression of endometrial pathological biomarkers including ER, PR, PRB and Ki-67 was also assessed by immunohistochemistry.

**Results:**

For the 53 recruited patients, overall complete response, recurrence and pregnancy rates were 94%, 26% and 36.4%. During the treatment, the serum biomarkers of HOMA-IR remained stable, while pathological markers including PR, PRB and Ki67 diminished significantly. Patients who achieved remission faster had significant lower HOMA-IR level and higher PRB expression at baseline. We also found a more remarkable down-regulation of PRB related with faster remission. Further multivariate analysis confirmed that baseline HOMA-IR ≥ 2.5 negatively affected treatment time to remission (OR 0.206; p = 0.017). While marked reduction of PRB (≥ 30%) at 3–6 months' treatment correlated with faster remission (OR 5.788; p = 0.010).

**Conclusion:**

For EC and EAH patients who received fertility-sparing therapy, baseline status of insulin resistance predicted poor response to progestin, while marked reduction of PRB following the initial 3–6 months' treatment predicted fast remission.

**Supplementary Information:**

The online version contains supplementary material available at 10.1186/s12905-021-01383-5.

## Background

The incidence of Endometrial cancer (EC) is rising in China in recent years, with the increasing prevalence of metabolic syndrome. As young patients tend to postpone delivery, more patients will face the contradictory situation of cancer treatment and uterus preservation. Many studies have proven the efficacy and safety of progestin-based fertility-sparing therapy for patients with EC or endometrial atypical hyperplasia (EAH), with a relatively high neoplasm remission rate of 75.3–88.7% [1].

Excessive unopposed exposure of endometrium to estrogen has long been regarded as the most important risk factors for endometriod cancer [2]. Additional risk factors include abnormal glycometabolism, insulin resistance (IR) and thyroid disease [3–5]. But the influence of such serum biomarkers on treatment efficacy hasn't been fully discussed under the fertility-sparing situation. Well-differentiated EC also pathologically presents with positive expression of estrogen receptor (ER) and progesterone receptor (PR) associated with good prognostic [6]. But it's unclear how such pathological biomarkers change during the conservative treatment. We neither know whether their expression or changed status relates with therapeutic response.

This study analyzed the dynamic change of serum and pathological biomarkers and explored their relationship with treatment outcomes in order to find efficacy predictive markers for fertility-sparing therapy of EC or EAH.

## Methods

### Eligibility

This was a retrospective cohort study of women who received fertility-sparing therapy for EC or EAH between January 2012 and December 2016 at Peking University People’s Hospital. The protocol for this study was approved by the Independent Ethics Committee (IEC) of Peking University People’s Hospital (Approval Number: 2016PHB054-01). Informed consent was obtained from all the patients recruited.

### Study design

**Fig. 1:**
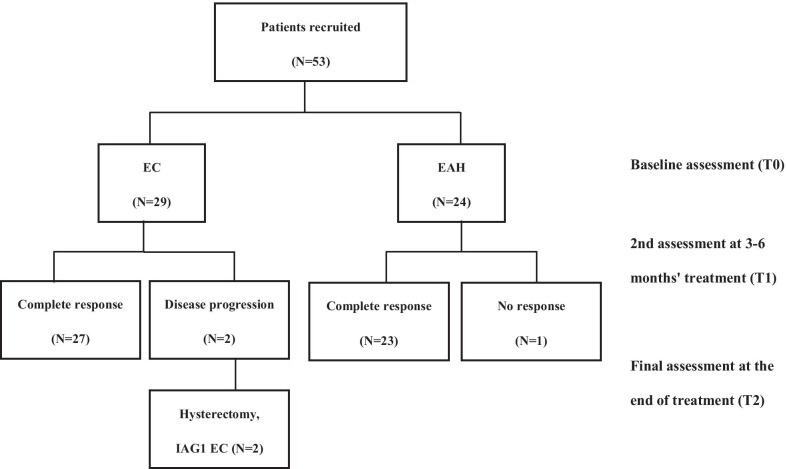
Study design

Study design is provided in Fig. 1. Patients who met the NCCN guidelines for fertility-sparing treatment of EC or EAH were recruited. Patients were treated with medroxyprogesterone (MPA), megestrol acetate (MA), or in combined with gonadotropin-releasing hormone agonist (GnRHa). Endometrial sampling was performed by hysteroscopy every three to six months during treatment to evaluate the histologic response. Pathology was assessed by senior gynecological histopathologists, using the WHO classification system [7].

Serum and endometrial tissue were obtained from each patient at three time points (T0, T1 and T2). The first point (T0) was to detect the serum and pathological biomarkers prior to treatment, which represents the baseline status. The second (T1) represented the assessment after 3–6 months of treatment. And the third (T2) represented the last assessment at the end of conservative treatment.

### Serum biomarker quantification

We measured serum biomarkers as follows: (a) sex hormones included the levels of luteinizing hormone (LH), follicle-stimulating hormone (FSH), estradiol (E), testosterone (T) and prolactin (PRL); (b) the status of IR which was evaluated by fasting blood glucose and insulin levels to derive the homeostasis model assessment of insulin resistance (HOMA-IR) and the glycosylated hemoglobin A1c (HbA1c) level; and (c) thyroid function included the levels of free thyroxine and thyroid-stimulating hormone (TSH). Individuals were classified as insulin resistant if HOMA-IR ≥ 2.5 [8].

### Pathological response evaluation and biomarker quantification

**Fig. 2:**
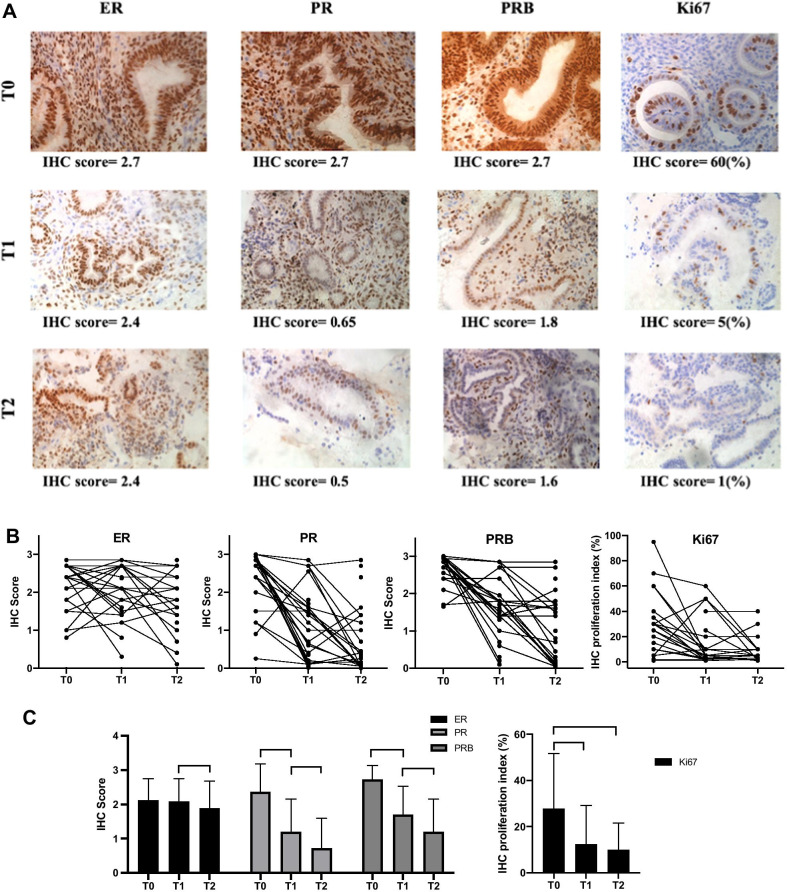
Changes in pathological markers at three time points during treatment. **A** Representative IHC images of ER, PR, PRB and Ki67 in sequential endometrial biopsy samples at baseline (T0), at 3–6 months' treatment (T1) and at the end of treatment (T2) from one patient. **B** IHC scores of above markers at T0, T1 and T2, are presented as scatter plots using paired data. **C** Median IHC score values at three time points based on data in panels** A**. * p < 0.05, ** p < 0.01

The response to progestin treatment was assessed histologically as follows: complete response (CR) was defined as a lack of residual EAH or EC; partial response (PR) was defined as a histological downgrade during follow-up; no response (NR) was defined as no evidence of disease regression or progression; and progression of disease (PD) was defined as a histological upgrade or if myometrial or extrauterine invasion was found during follow-up. Immunohistochemical (IHC) staining of pathological biomarkers included proliferation marker Ki-67 and hormone receptors, thus ER, PR and isoform B of progesterone receptor (PRB). Staining for Ki-67 was performed according to the number of positive staining nuclei per high-power field. The product IHC score (0–3) for ER, PR, and PRB was calculated as the intensity of intranuclear staining (0, 1, 2, and 3) multiplied by the proportion of nuclear staining (0–1). Representative sequential images of IHC staining and IHC scores for these four pathological biomarkers at three time points are shown in Fig. 2.

### Statistical analysis

We analyzed the changes of serum and pathological biomarkers during the three time points (T1 vs. T0, T2 vs. T0), to describe the variation trend. Then we compared above biomarkers and their changes in different groups based on remission time, to find potential efficacy-related risk factors. We further use multivariate analysis to identify independent factors for predicting remission time.

For continuous data, normally distributed variables are expressed using the mean ± SD; nonnormally distributed variables are expressed using the median value (interquartile range). The strength of the difference between intergroup variables were determined using the Student's *t*-test or the Mann–Whitney *U* test. Intergroup differences for categorical variables were tested for significance using the chi-square test. Changes of biomarkers between paired time points (T1 vs. T0, T2 vs. T0) were determined using Wilcoxon signed-rank test. Variables yielding p values lower than 0.1 by univariate analysis were entered into a multivariate cox proportional hazard regression model to determine variables independently associated with treatment time to remission. All statistical tests were two sided at the 5% level of significance and were performed using SPSS Version 19.0 for Windows (IBM Corp, Armonk, NY, USA).

## Results

### Baseline characteristics

Study design was provided in Fig. 1. A total of 53 eligible patients, including 29 with EC and 24 with EAH, who received fertility-sparing treatment were recruited. Baseline demographic characteristics were shown in Additional file 1: Table 1. Patients were followed up from the end of conservative treatment until January 1, 2019. The median follow-up time was 46 months (23.5–78 months).

Oral progestin therapy, including MPA and MA, was used in 44 patients and 9 patients was treated with GnRHa alone or combined with oral progestin. Metformin was given to 20 (37.7%) patients if they had IR or at the discretion of the doctors. After remission, 37 (72.5%) patients were given maintenance therapy.

### Oncological and reproductive results

The overall CR rate was 94% (50/53). In addition, two patients with EC experienced PD, and one patient with EAH experienced NR. Among the 50 patients who achieved CR, 13 (26%) experienced recurrence, including 9 with EC and 4 with EAH. The time interval from CR to recurrence was 13 (8, 47) months.

Forty-four patients who achieved CR had a plan for parenthood. In total, 36.4% became pregnant (21 pregnancies). There were 38.6% (17/44) of patients who took assisted reproductive technology (ART). The live birth rate was 27.3% (12/44).

### Changes in serum biomarkers and their relationship with the treatment time to remission

During conservative treatment, serum biomarker levels were assessed at three time points, as shown in Additional file 1: Table 2. As expected, after progestin-based treatment, there was a decrease tendency in estradiol and a significant increase in FSH level. The level of LH decreased at the first follow-up and further decreased at the end of treatment. The level of testosterone also decreased at both the first and last follow-ups. There was no difference in the prolactin level during treatment.

Regarding glycometabolism, there were no significant changes in HbA1c or HOMA-IR after progestin treatment, nor were there any differences in the biomarkers of thyroid function (FT4, TSH).

We then compared the hormone markers in in patients who got remission within 6 months vs. those who did not (Table 1). In the "Remission time ≥ 6 months" group, we detected a significant higher baseline HOMA-IR level (4.2 vs. 2.2, p = 0.004). While patients in this group also showed a more remarkable decrease in the HOMA-IR level during progestin treatment (T1 vs. T0, -39% vs. -12%, p = 0.049; T2 vs. T0, -44% vs. 25%, p = 0.020). Further analysis showed that 41.9% (13/31) of patients in the "Remission time ≥ 6 months" were given metformin as a combination therapy, while only 26.3% (5/19) of patients in the "Remission time < 6 months" group. Other biomarkers indicating gonadal and thyroid hormones showed no significant difference between these two groups.

**Table 1:** Changes in serum markers in patients with different treatment time to remission

	Remission time < 6 months (n = 19)	Remission time ≥ 6 months (n = 31)	p-value
T0-Estradiol, pg/mL	105.2 ± 187.2	53.1 ± 83.9	0.322
T0-FSH, IU/L	5.5 ± 2.3	5.3 ± 3.1	0.831
T0-LH, IU/L	3.0 ± 2.1	3.8 ± 3.2	0.494
T0-T, nmol/L	1.0 ± 0.6	0.9 ± 0.7	0.672
T0-PRL, ng/mL	20.3 ± 11.2	21.0 ± 9.6	0.437
T0-HOMA-IR	2.2 ± 1.3	4.2 ± 2.0	**0.004**
T1-HOMA-IR	2.4 ± 1.5	3.6 ± 3.7	0.311
T2-HOMA-IR	2.7 ± 1.8	4.0 ± 2.9	0.185
T1 vs. T0-HOMA-IR	− 12%	− 39%	**0.049**
T2 vs. T0-HOMA-IR	25%	− 44%	**0.020**
T0-HbA1c, %	5.7 ± 0.4	5.6 ± 0.4	0.922
T0-FT4, pmol/L	16.4 ± 2.8	16.8 ± 1.9	0.688
T0-TSH, uIU/mL	2.1 ± 1.7	2.2 ± 0.9	0.864

Bold part represents p-value less than or near 0.05

E, estradiol; FSH, follicle-stimulating hormone; LH, luteinizing hormone; T, testosterone; PRL, prolactin; HOMA-IR, homeostasis model assessment of insulin resistance; HbA1c, glycosylated hemoglobin A1c; FT4, free thyroxine; TSH, thyroid-stimulating hormone

### Changes in pathological biomarkers and their relationship with the treatment time to remission

We first examined hormone receptor expression in paired endometrial biopsies obtained at three time points (Fig. 2). Most patients demonstrated a significant reduction in PR and PRB proteins expression post-treatment as compared to baseline (T1 vs. T0) (Fig. 2B,C). There is also further significant reduction at the final assessment in PR and PRB proteins (T2 vs. T1) (Fig. 2C). The ER status remained stable during the first 3–6 months of treatment, while showed a slight decrease at the end of treatment (Fig. 2C). There is also significant reduction of Ki67 protein during the treatment (Fig. 2B, C).

**Fig. 3:**
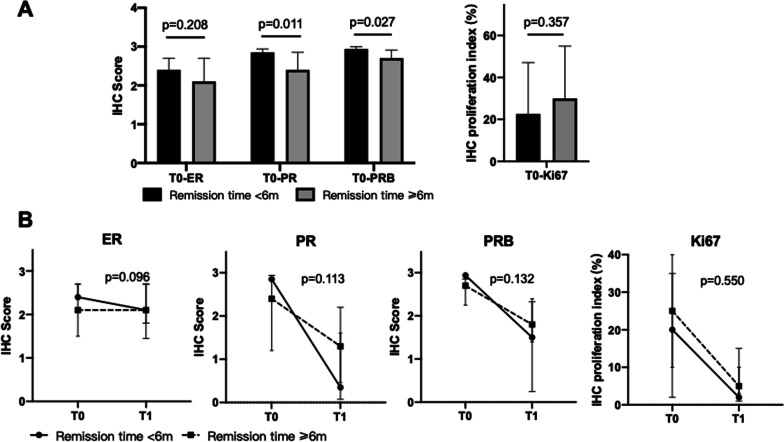
Changes in pathological markers in patients with different treatment time to remission (< 6 months vs. ≥ 6 months). **A** Comparison of median IHC score values of ER, PR, PRB and Ki67 proteins at baseline assessment in patients that regressed < 6 months vs. ≥ 6 months. **B** Comparison of median change of IHC scores at 3–6 months treatment (T1) compared to baseline level (T0) in patients that regressed < 6 months vs. ≥ 6 months

We also compared hormone receptor levels in patients who got remission within 6 months vs. those who did not (Fig. 3). In the “Remission time < 6 months” group, we found significant higher baseline PR, and PRB proteins (Fig. 3A). While the baseline ER and Ki67 levels showed no significant difference (Fig. 3A). Furthermore, patients in the "Remission time < 6 months" also showed a tendency of greater downregulation of ER (− 41% vs. 0, p = 0.096), PR (− 88% vs. − 58%, p = 0.113) and PRB (− 47% vs. − 33%, p = 0.132) in the second assessment compared to the baseline level (Fig. 3B). While the changes of Ki67 protein between the two groups showed no difference (Fig. 3B).

### Independent risk factors for predicting treatment time to remission

We next analyzed the risk factors for predicting treatment time to remission (Table 2). The univariate analysis showed that age ≥ 30 years (p = 0.063) and HOMA-IR ≥ 2.5 (p = 0.029) seemed negatively correlate with time to remission. The pathology of EAH compared to EC (p = 0.011) positively correlated with time to remission. Also, marked down-regulation of PRB (≥ 30%) at the second assessment (p = 0.019) positively correlated with treatment time to remission. While we didn't detect significant relations between patients' remission time and BMI, whether combined with T2DM, PCOS, thyroid disease, usage of metformin, or baseline level of pathological biomarkers.

**Table 2:** Cox analysis of factors associated with treatment time to remission

		Univariate analysis		Multivariate analysis	
		OR (95% CI)	p-value	OR (95% CI)	p-value
BMI (kg/m^2^)	≥ 25	0.717 (0.406–1.268)	0.253		
	< 25	1			
Age	≥ 30	**0.544 (0.286–1.032)**	**0.063**	**–**	**–**
	< 30	**1**			
Pathology	EAH	**2.120 (1.186–3.789)**	**0.011**	**3.870 (1.175–12.742)**	**0.026**
	EC	1		1	
T2DM	Yes	0.816 (0.427–1.957)	0.914		
	No	1			
PCOS	Yes	0.916 (0.476–1.764)	0.793		
	No	1			
Hypothyroidism	Yes	1.842 (0.868–3.908)	0.111		
	No	1			
Metformin	Yes	0.626 (0.324–1.210)	0.164		
	No	1			
HOMA-IR	≥ 2.5	**0.440 (0.210–0.921)**	**0.029**	**0.206 (0.056–0.755)**	**0.017**
	< 2.5	**1**		**1**	
T0-ER		1.461 (0.676–3.158)	0.335		
T0-PR		1.198 (0.651–2.205)	0.561		
T0-PRB		2.560 (0.676–9.695)	0.167		
T1 vs. T0-ER*	≥ 0	1.397 (0.599–3.256)	0.439		
	< 0	1			
T1 vs. T0-PR*	≥ 60%	1.223 (0.504–2.971)	0.656		
	< 60%	1			
T1 vs. T0-PRB*	≥ 30%	**2.995 (1.201–7.469)**	**0.019**	**5.788 (1.531–21.889)**	**0.010**
	< 30%	**1**		**1**	
T0-Ki67		1.002 (0.986–1.018)	0.826		

Bold part represents p-value less than or near 0.05

*Defined as the range of reduction at the second assessment compared to the baseline level. OR: odds ratio; CI, confidence interval; EC, endometrial cancer; EAH, endometrial atypical hyperplasia; BMI, body mass index; T2DM, type 2 diabetes mellitus; PCOS, polycystic ovary syndrome

After adjusting for age, the further multivariate analysis demonstrated only pathological type, HOMA-IR level and marked reduction of PRB were the independent factors related with treatment time to remission (Table 2). Patients with a reduction in PRB (≥ 30%) at 3–6 months' treatment, significantly correlated with faster remission (odds ratio [OR], 5.788 95% CI 1.531–21.889 p = 0.010). While those with insulin resistance status (HOMA-IR ≥ 2.5) and those with a histological type of EC (compared with EAH) both negatively affected treatment time to remission (OR 0.206 95% CI 0.056–0.755 p = 0.017; OR 0.206 95% CI 0.056–0.755 p = 0.017, respectively).

## Discussion

The objective of this study was to determine the changes in expression of serum and pathological biomarkers during progestin therapy for EAH and EC patients and what implications in prediction of treatment efficacy would be expected.

Our study found, no significant change in HbA1c or HOMA-IR during fertility-sparing treatment, which implies such a dose of progestin does not aggravate or alleviate the status of insulin resistant or glycometabolism for patients. Our results also indicated that a higher baseline level of HOMA-IR related to a longer remission time. However, for patients with longer remission time their HOMA-IR level tended to decrease following treatment than those with faster remission (− 44% vs. 25%, p = 0.020). This might be due to the combined use of metformin and weight loss, as often suggested by doctors for patients who had initial insulin resistance. The result also proved that more patients in this group used metformin (41.9% vs. 26.3%) and their status of insulin resistance has an expectant improvement.

Further multivariate Cox analysis confirmed that patients with HOMA-IR ≥ 2.5 correlated with longer remission time. Insulin resistance has long been regarded as an important risk factor for the development of EC [9, 10]. However, few reports have analyzed the role of IR in fertility-sparing treatment. A review showed diabetes mellitus (DM) seems not affect the outcome of conservative treatment in EAH and EC, though the number of included patients diagnosed with DM is small [11]. Yang [12] analyzed EAH patients and found that, IR significantly affected the treatment time to remission (8.1 months with IR vs. 6.1 months without IR, p = 0.004). Our result also proved that for EC and EAH patients, IR status prolonged the treatment time. We propose IR might negatively affect the treatment response to progestin through several mechanisms. Firstly, insulin could act directly on EC cells through the PI3K/AKT/mTOR signaling pathway that promotes cell survival and proliferation [13–15]. Secondly, insulin resistance is described to suppress hepatic sex hormone-binding globulin production and induce ovarian steroid hormone production which result in increased bioavailable estrogen and alleviates the antiestrogen effect [16]. Additionally, insulin could induce cholesterol synthetase DHCR24 expression which inversely correlates with PR and thus aggravating progestin resistance in EC cells [17]. Therefore, we believe insulin resistance would compromise the therapeutic effect of progestin and thus prolong the treatment time. For such patients with IR, it's reasonable to treat them more than 6 months or even longer. On the other hand, we should consider the benefit of the combined use of metformin to improve their IR status which might potentially improve the therapeutic efficacy.

Regarding the pathological markers, we found that the levels of PR and its subtype PRB diminished post-treatment, and patients with marked downregulated levels of PRB at the 3–6 months of treatment predicted faster remission. This might be because a downregulation of PRB in good responders could be indicative of active receptors which is usually ubiquitinated and turned over rapidly. For now, reports regarding the utility of hormone receptor expression in predicting the response to progestin-based therapy have been controversial [18]. In consistent with our finding, Vereide’s research in endometrial hyperplasia (EH) patients showed that PR, ER or their isoforms were downregulated in responders and stably expressed in nonresponders [19]. Raffone's retrospective study showed a weak stromal PRB expression is a highly sensitive predictive marker of no response of AEH and EEC conservatively treated [20]. Another small study of a 10-patient cohort found that ER and PRB levels after treatment with a progestin-containing IUD were significantly higher in the “progression” group than in the “no progression” group [21]. Also, a systematic review showed PR is significantly predictive of response in EH and EC treated with a levonorgestrel-intrauterine device, but not with oral progestins [22]. However, other studies did not report any significant associations [23, 24]. The GOG211 trial found that only low pre-treatment levels of the Ki-67 level were a predictor of the histologic response, rather than ER or PR [25]. Given the high remission rate to progestin therapy, the treatment time rather than the remission rate might contribute to above inconsistent results. Additionally, the small sample size of the above studies might have prevented a reliable conclusion to be drawn. Our results imply that greater change in PRB expression after initial treatment may serve as promising markers for early predicting response to conservative therapy.

There are several limitations to our study. First, retrospective data collection might produce bias with information ascertainment. Second, our relatively small sample size reduces the ability to draw more convincing conclusions. A large-sample prospective study is needed to verify our preliminary findings. Third, due to the limited number of patients, we could not investigate the effect of metformin on changes of serum or pathological biomarkers. This implication remains to be further demonstrated. However, this study described a comprehensive picture for the first time of changing trend in both endocrine hormone levels and pathological markers during fertility-sparing treatment. This study also represents one of the largest series in the literature which proved the significance of baseline insulin resistance and the marked change in PRB proteins in predicting treatment efficacy.

In conclusion, the current study shows that during fertility-sparing treatment for EC and EAH patients, insulin resistance status or thyroid function is not affected, while progesterone receptor level diminishes significantly. As for treatment efficacy, insulin resistant status of patients predicts longer treatment time to remission, while marked reduction of PRB after 3–6 months' treatment has good implications for faster remission.

## Supplementary Information

**Additional file 1**.** Supplementary Table 1**. Patient demographics.** Supplementary Table 2**. Changes in markers of insulin resistance, thyroid function and gonadal function at three time points during treatment.

## Data Availability

All data generated or analysed during this study are included in this published article and its supplementary tables. Dr. YQ Wang could be contacted if data from this study requested.

## References

[1] Qin Y, Yu Z, Yang J, Cao D, Yu M, Wang Y, Shen K (2016). Oral progestin treatment for early-stage endometrial cancer: a systematic review and meta-analysis. Int J Gynecol Cancer.

[2] Morice P, Leary A, Creutzberg C, Abu-Rustum N, Darai E (2016). Endometrial cancer. Lancet.

[3] Liao C, Zhang D, Mungo C, Tompkins DA, Zeidan AM (2014). Is diabetes mellitus associated with increased incidence and disease-specific mortality in endometrial cancer? A systematic review and meta-analysis of cohort studies. Gynecol Oncol.

[4] Raffone A, Travaglino A, Saccone G, D'Alessandro P, Arduino B, Mascolo M, De Placido G, Insabato L, Zullo F (2020). Diabetes mellitus is associated with occult cancer in endometrial hyperplasia. Pathol Oncol Res.

[5] Wang Y, Zhou R, Wang J (2019). Relationship between hypothyroidism and endometrial cancer. Aging Dis.

[6] Jerzak KJ, Duska L, MacKay HJ (2019). Endocrine therapy in endometrial cancer: an old dog with new tricks. Gynecol Oncol.

[7] Kurman R, Carcangiu M, Herrington C, Young R, Kurman R, Carcangiu M, Herrington C, Young R: WHO Classification of tumors of female reproductive organs. 2014.

[8] Mitsuhashi A, Habu Y, Kobayashi T, Kawarai Y, Ishikawa H, Usui H, Shozu M (2019). Long-term outcomes of progestin plus metformin as a fertility-sparing treatment for atypical endometrial hyperplasia and endometrial cancer patients. J Gynecol Oncol.

[9] Giovannucci E, Harlan DM, Archer MC, Bergenstal RM, Gapstur SM, Habel LA, Pollak M, Regensteiner JG, Yee D (2010). Diabetes and cancer: a consensus report. Diabetes Care.

[10] Shikata K, Ninomiya T, Kiyohara Y (2013). Diabetes mellitus and cancer risk: review of the epidemiological evidence. Cancer Sci.

[11] Raffone A, Travaglino A, Saccone G, Di Maio A, Mollo A, Mascolo M, De Rosa R, De Placido G, Insabato L, Zullo F (2019). Diabetes mellitus and responsiveness of endometrial hyperplasia and early endometrial cancer to conservative treatment. Gynecol Endocrinol.

[12] Yang B, Xie L, Zhang H, Zhu Q, Du Y, Luo X, Chen X (2018). Insulin resistance and overweight prolonged fertility-sparing treatment duration in endometrial atypical hyperplasia patients. J Gynecol Oncol.

[13] Wang C-F, Zhang G, Zhao L-J, Qi W-J, Li X-P, Wang J-L, Wei L-H (2013). Overexpression of the insulin receptor isoform A promotes endometrial carcinoma cell growth. PLoS ONE.

[14] Schmandt RE, Iglesias DA, Co NN, Lu KH (2011). Understanding obesity and endometrial cancer risk: opportunities for prevention. Am J Obstet Gynecol.

[15] Bartella V, De Marco P, Malaguarnera R, Belfiore A, Maggiolini M (2012). New advances on the functional cross-talk between insulin-like growth factor-I and estrogen signaling in cancer. Cell Signal.

[16] Le TN, Nestler JE, Strauss JF, Wickham EP (2012). Sex hormone-binding globulin and type 2 diabetes mellitus. Trends Endocrinol Metab.

[17] Dai M, Zhu X-L, Liu F, Xu Q-Y, Ge Q-L, Jiang S-H, Yang X-M, Li J, Wang Y-H, Wu Q-K (2017). Cholesterol synthetase DHCR24 induced by insulin aggravates cancer invasion and progesterone resistance in endometrial carcinoma. Sci Rep.

[18] Travaglino A, Raffone A, Saccone G, Insabato L, Mollo A, De Placido G, Zullo F (2019). Immunohistochemical predictive markers of response to conservative treatment of endometrial hyperplasia and early endometrial cancer: a systematic review. Acta Obstet Gynecol Scand.

[19] Vereide AB, Kaino T, Sager G, Arnes M, Ørbo A (2006). Effect of levonorgestrel IUD and oral medroxyprogesterone acetate on glandular and stromal progesterone receptors (PRA and PRB), and estrogen receptors (ER-alpha and ER-beta) in human endometrial hyperplasia. Gynecol Oncol.

[20] Raffone A, Travaglino A, Zullo FM, Gencarelli A, Micheli M, Miranda S, De Franciscis P, Insabato L, Di Spiezio Sardo A, Zullo F *et al*: Predictive Accuracy of progesterone receptor B in young women with atypical endometrial hyperplasia and early endometrial cancer treated with hysteroscopic resection plus LNG-IUD Insertion. *J Minim Invasive Gynecol* 2020. 10.1016/j.jmig.2020.10.009 33122144

[21] Reyes HD, Carlson MJ, Devor EJ, Zhang Y, Thiel KW, Samuelson MI, McDonald M, Yang S, Stephan J-M, Savage EC (2016). Downregulation of FOXO1 mRNA levels predicts treatment failure in patients with endometrial pathology conservatively managed with progestin-containing intrauterine devices. Gynecol Oncol.

[22] Raffone A, Travaglino A, Saccone G, Mollo A, De Placido G, Insabato L, Zullo F (2019). Should progesterone and estrogen receptors be assessed for predicting the response to conservative treatment of endometrial hyperplasia and cancer? A systematic review and meta-analysis. Acta Obstet Gynecol Scand.

[23] Gunderson CC, Dutta S, Fader AN, Maniar KP, Nasseri-Nik N, Bristow RE, Diaz-Montes TP, Palermo R, Kurman RJ (2014). Pathologic features associated with resolution of complex atypical hyperplasia and grade 1 endometrial adenocarcinoma after progestin therapy. Gynecol Oncol.

[24] Yang Y-F, Liao Y-Y, Liu X-L, Su S-G, Li L-Z, Peng N-F (2015). Prognostic factors of regression and relapse of complex atypical hyperplasia and well-differentiated endometrioid carcinoma with conservative treatment. Gynecol Oncol.

[25] Zaino RJ, Brady WE, Todd W, Leslie K, Fischer EG, Horowitz NS, Mannel RS, Walker JL, Ivanovic M, Duska LR (2014). Histologic effects of medroxyprogesterone acetate on endometrioid endometrial adenocarcinoma: a Gynecologic Oncology Group study. Int J Gynecol Pathol.

