# Supplementary material for: Significance of serum and pathological biomarkers in fertility-sparing treatment for endometrial cancer or atypical hyperplasia: a retrospective cohort study
Source: BMC Womens Health. 2021 Jun 23;21:252. doi: 10.1186/s12905-021-01383-5 (PMC8223344; doi:10.1186/s12905-021-01383-5)
Supplement: Supplementary file 1 — Additional file 1. Supplementary Table 1. Patient demographics. Supplementary Table 2. Changes in markers of insulin resistance, thyroid function and gonadal function at three time points during treatment. [file 12905_2021_1383_MOESM1_ESM.docx]

**Supplementary Table 1.** **Patient demographics**

| Pathological type | | All (n=53) |
| --- | --- | --- |
| Age at diagnosis | | 31±5 |
| Nulliparous |  | 48 (90.6%) |
| BMI (kg/m^2^) | | 26.7 ±5.9 |
| WHR | | 0.87±0.08 |
| Systolic pressure (mmHg) | | 121±12 |
| Diastolic pressure (mmHg) | | 78±9 |
| T2DM | | 9 (17%) |
| PCOS | | 12 (22.6%) |
| Hypothyroidism | | 9 (17%) |
| Family malignant tumor history | | 2 (3.8%) |

BMI, body mass index; WHR, Waist-to-hip ratio; T2DM, type 2 diabetes mellitus; PCOS, polycystic ovary syndrome

**Supplementary Table 2. Changes in markers of insulin resistance, thyroid function and gonadal function at three time points during treatment**

|  | T0 (n=31) | | T1 vs. T0 (n=27) | |  | T2 vs. T0 (n=19) | |
| --- | --- | --- | --- | --- | --- | --- | --- |
|  |  |  | Mean difference  (95% CI) | p-value |  | Mean difference  (95% CI) | p-value |
| Estradiol, pg/mL | 30.0 (12.1, 60.9) | -51.2 (-107.8, 5.3) | | **0.074** |  | -36.1 (-95.9, 23.8) | 0.221 |
| FSH, IU/L | 5.3 (3.7, 6.2) | 1.0 (0.1, 2.0) | | **0.038** |  | 1.0 (0.4, 2.2) | 0.158 |
| LH, IU/L | 2.7 (1.5, 5.2) | -1.8 (-2.7, -0.9) | | **0.000** |  | -2.4 (-3.8, -1.0) | **0.002** |
| T, nmol/L | 0.9 (0.2, 1.4) | -0.5 (-0.7, -0.3) | | **0.000** |  | -0.4 (-0.7, -0.0) | **0.044** |
| PRL, ng/mL | 18.9 (13.5, 27.9) | 2.9 (-3.5, 9.3) | | 0.362 |  | 1.6 (-5.3, 8.5) | 0.626 |
| HbA1c, % | 5.6 (5.3, 5.8) | 0.1 (-0.1, 0.2) | | 0.464 |  | 0.1 (-0.1, 0.3) | 0.472 |
| HOMA-IR | 2.9 (2.0, 4.6) | -0.5 (-1.6, 0.6) | | 0.368 |  | -0.6 (-2.0, 0.8) | 0.396 |
| FT4, pmol/L | 16.6 (15.2, 18.4) | -0.6 (-1.8, 0.6) | | 0.327 |  | 0.0 (-1.3, 1.3) | 0.978 |
| TSH, uIU/mL | 1.9 (1.4, 2.8) | 0.2 (-0.5, 0.8) | | 0.535 |  | -0.2 (-1.1, 0.8) | 0.696 |

CI, confidence interval; E, estradiol; FSH, follicle-stimulating hormone; LH, luteinizing hormone; T, testosterone; PRL, prolactin; HbA1c, glycosylated hemoglobin A1c; HOMA-IR, homeostasis model assessment of insulin resistance; FT4, free thyroxine; TSH, thyroid-stimulating hormone
